# Supplementary material for: Acceptability and Feasibility of Maternal Mental Health Assessment When Managing Small, Nutritionally At-Risk Infants Aged < 6 Months: A Key Informant Interview Study
Source: Children (Basel). 2024 Feb 6;11(2):209. doi: 10.3390/children11020209 (PMC10887604; doi:10.3390/children11020209)
Supplement: Supplementary file 1 [file children-11-00209-s001.zip › children-2839874-supplementary.pdf]

Pre-interview questions:

- Can you tell me a bit about your role? Are you:
    - Health Practioner/Implementer (e.g. nurse, doctor, clinical psychologist, dietitian or other Allied Health Professional?)
    - Researcher
    - Policy maker

- What region do you currently work in?
    - West Africa
    - East Africa
    - South Africa
    - South Asia
    - International (i.e. more than 1 region)
    - Other (if so, where …..)

- How many years of field experience do you have?
    - 0-5
    - 5-10
    - 10+

- Have you had any formal mental health training in your undergraduate or post-graduate studies?
    - No
    - Yes
        - If so, how long was the training?
            - 1 hour
            - A single morning/afternoon
            - A full day
            - More than 1 day
            - Unsure

- Have you had any formal mental health training since gaining your degree?
    - No
    - Yes
        - If so, how many years post-education did this training take place?
            - 0-5
            - 5-10
            - 10-15
            - 15+
            - Unsure

- Have you any experience of using a validated maternal mental health screening tool?
    - For example:
        - Patient Health Questionnaire (PHQ-9)
        - Edinburgh Postnatal Depression Screen
        - Beck's Depression Inventory (BDI)
        - The Mothers Object Relations Scales (MORS)

- Have you been involved in the development of the MAMI care pathway?
    - Yes or No

Questions:

Q1. I wondered if you could tell me about your experience, or your colleagues/teams' experiences, of assessing maternal mental health when managing nutritionally at-risk infants? This could be in clinical practice or from a decision-making perspective (e.g. where to direct funds, applying guidelines).

a. Can you tell me more about this (negative/positive) situation and the subsequent impacts of this on your approach to maternal mental health assessments?

Q2. Can you tell me about any challenges you have faced when assessing maternal mental health, or that you would expect to face in your context?

a. Do you have any examples from your staff or team members?
b. Have you experienced any challenges regarding practicality/service availability for treatment/logistical/staffing (training and numbers)/interpretation of questions (from staff and mothers)?

Q3. What is your experience or understanding of how acceptable assessing, discussing and treating mental health is within the communities you work with?

a. How do you envision mental health assessments for mothers, especially those with nutritionally vulnerable children, being perceived and accepted by the communities you work with?
b. And, generalising, how about the staff's perceptions?

Q4. What, if any, stigmas exist regarding a mental health diagnosis for mothers in your setting?

a. Are there any stigmas regarding maternal mental health treatments?
b. What treatments exist in your setting currently, if any?

Q5. Has there ever been a time you have omitted questions on a screening tool?

a. What were the reasons why you did/did not do this?
b. There is debate around asking mothers about suicidal intent when a service has sub-optimal treatment options; what are thoughts on this?

Q6. What are your thoughts about the feasibility of maternal mental health assessments in the communities you work with?

a. Are there any risks of assessing maternal mental health to the infants?
b. And what about the wider family unit? And the patient-facing staff?

c. Are there any risks of asking about mental health when a service does not have sufficient treatment provision to support a mother with mental health difficulties?

Q7. What, if any, are the benefits of assessing maternal mental health in the communities you work with?

a. Are there any benefits of assessing maternal mental health to the infants?
b. And what about the wider family unit? And the patient-facing staff?
c. Are there any benefits of asking about mental health when a service does not have sufficient treatment provision to support a mother with mental health difficulties?
    i. Does simply asking and talking to mothers, regardless of service provision, still help?

Q8. Can you tell me about how you think maternal mental health assessments could be implemented, or improved, in your context, if you feel they could?

b. And why? What factors would hinder/help implementation?
c. What staff training and/or health systems are needed, if any?
d. Can non-specialist staff and non-specialist teams conduct mental health assessments in your setting?
    i. In a context with no specialist services, to what extent are routine services supporting and/or addressing maternal mental health?
e. What are the tangible next steps, over the next 2-5 years, for implementation or improvement of maternal mental health services in your context?

End of interview – thank you for your time and for sharing your thoughts and experiences with me today. Before we end, was there anything else you wanted to add to the questions you've answered, or new themes you want to discuss?
